# Two-Dimensional DOA Estimation of Coherent Sources Based on Uniform Linear Electromagnetic Vector Sensor Array

**DOI:** 10.3390/s26092829

**Published:** 2026-05-01

**Authors:** Jingxiang Zhang, Xiang Lan, Xianpeng Wang

**Affiliations:** School of Information and Communication Engineering, Hainan University, Haikou 570228, China; zhangjx@hainanu.edu.cn (J.Z.); wxpeng2016@hainanu.edu.cn (X.W.)

**Keywords:** 2D-DOA estimation, coherent signal, linear tripole array, Toeplitz matrix reconstruction

## Abstract

In this paper, the two-dimensional DOA estimation problem of coherent signals in an electromagnetic vector sensor (EMVS) array is studied. A new decorrelation method is proposed by extending the multiple-Toeplitz matrices reconstruction (MTOEP) method to a polarization sensitive array. After that, a closed-form solution is derived based on the ESPRIT algorithm for DOA and polarization parameter estimation. Pairing matching is based on the correspondence between eigenvectors obtained by independent eigen-decomposition. Simulation results verify the effectiveness of the proposed work and show the proposed algorithm has better performance than the traditional spatial smoothing (SS) method, especially in scenarios with low signal-to-noise ratios (SNRs) and small angular separations.

## 1. Introduction

The estimation of two-dimensional direction of arrival (2D-DOA) is essential in the field of array signal processing, and has been widely applied in military and civilian uses such as radar, sonar and wireless communication. After decades of development, many high-resolution methods are studied, including subspace-based algorithms [1,2,3], sparse recovery (SSR) algorithms [4,5,6,7] and tensor-based algorithms [8,9,10]. As the sensing distance decreases and scenarios become more diverse and complex in modern sensing systems, researchers are no longer limited to the idealized assumption of far-field target localization. As a result, extensive studies have been conducted on near-field DOA estimation and mixed near-field and far-field localization problems [10,11]. Furthermore, under challenging conditions such as signal coherency and underdetermined observations, limitations of the DOA estimation methods relying solely on second-order statistics have become increasingly evident. To further exploit the information embedded in array measurements, researchers have explored third-order and higher-order cumulant-based approaches, along with tailored array design strategies, to further enhance the degrees of freedom [12,13]. However, most algorithms studied focus on a scalar sensor array, where sensors receive signals omnidirectionally in space. As an inherent property of electromagnetic waves, polarization makes incident signals vary along the receiving direction. A vector sensor array is proposed to receive the signal difference due to the polarization in order to achieve better performance [14]. Compared with traditional scalar sensor, a electromagnetic vector sensor (EMVS) collects more information of incident signals with better anti-interference ability, stronger detection ability, and higher system resolution. Normally, a full EMVS has six components including three dipoles and three loops co-located for measuring the electric and magnetic field of a signal, respectively. However, compared with a scalar sensor that has only one component, having more receiving components also leads to higher data dimensionality, which leads to a significant increase in computational complexity in signal processing [15,16]. An alternative solution is to reduce the components of the EMVS. At present, most research is still based on a crossed-dipole sensor array [17], and X. Lan has theoretically proven that a uniform linear array (ULA) with tripole sensors can achieve 2D-DOA estimation [18]. Compared with the crossed-dipole sensor, the tripole can obtain complete electric field information, and a tripole array can exploit the quantitative relationship between polarization in three different directions. Therefore, the research is studied on a ULA with a tripole sensor.

Most algorithms assume that signals are independent of each other. But in practice, there exist coherent signals in received signals due to multipath effects or electronic countermeasures. The coherency between signals results in the inability to properly separate the signal subspace and the noise subspace, making it difficult for subspace-based algorithms based on eigen-decomposition to accurately estimate DOA and polarization parameters of incident signals. To solve this problem, J. Li applied the spatial smoothing (SS) algorithm to EMVS arrays and achieved DOA estimation with the ESPRIT algorithm [19]. SS correctly resolves the separation between the signal subspace and the noise subspace, but it causes a reduction in the effective array aperture due to subarray partitioning. In addition, the maximum number of coherent signals that can be handled is limited by smoothing times [20]. Rahamim proposed and conducted many studies on the polarization smoothing algorithm (PSA) [21]. The PSA is not limited by array structure without loss of array aperture, making it applicable to arbitrary-shaped arrays. But it ruins the structure of polarization steering vectors and cannot continue following polarization parameter estimation [22]. In an acoustic vector sensor array, Palanisamy and Liu handled the rank-deficit issue by constructing structured matrices based on cross-correlation between subarrays to achieve decorrelation in an L-shaped array [23] and a uniform rectangular array [24], respectively. In scenarios where coherent and non-coherent signals coexist, Molaei did not explicitly resolve the rank-deficit issue, but instead proposed a clustering-based efficient separation method to distinguish the two types of signals, enabling subsequent DOA estimation to be performed separately [25]. In addition, several non-subspace-based algorithms, such as subspace fitting algorithms [26] and sparse recovery algorithms [27,28,29], have also been employed to handle coherent signal scenarios, owing to their insensitivity to inter-signal coherency. To address the rank deficient problem, in addition to smoothing methods, another class of algorithms is the Toeplitz matrix reconstruction approach, which takes advantage of the fact that an ideal covariance matrix exhibits the Toeplitz–Hermitian property. By reconstructing a smaller-dimension, full-rank matrix that satisfies this property—using, for example, eigenvectors of the covariance matrix [30] or the received array data [31,32]—as an equivalent covariance matrix, more accurate DOA estimation can be achieved. However, these methods only utilize partial information from the covariance matrix.

Recently, a method called multiple-Toeplitz Hermitian matrices reconstruction (MTOEP) and its improvements have been proposed to restore the rank of the covariance matrix and achieve superior performance in estimation accuracy [33,34], which exploit the shift-invariance property of the array and the Toeplitz structure in an ideal covariance matrix. Compared with the previously mentioned algorithm [30,31], MTOEP does not require auxiliary vector sensors or denoising processing and can fully utilize all array outputs. This more comprehensive use of covariance information theoretically improves estimation accuracy. It is observed that these state-of-the-art algorithms have not yet been investigated or applied in vector sensor arrays, and their potential remains largely unexplored, suggesting considerable prospects for further study. In this paper, we combine the ESPRIT algorithm with the block multiple-Toeplitz matrix reconstruction (BMTOEP) method, and propose a new algorithm to overcome the difficulty of 2D-DOA estimation of coherent signals in a uniform linear tripole array. Firstly, a novel preprocessing approach based on block-Toeplitz matrix reconstruction is applied to remove the coherency between incident signals. Then, the array is partitioned into different subarrays twice to obtain three rotational invariant factors using the ESPRIT algorithm. Finally, unambiguous DOA and polarization parameters are estimated from the invariants.

The structure of this paper is as follows. The coherent signal model for the EMVS array is presented in Section 2 and the proposed method is developed in Section 3. In Section 4, we provide numerical simulation results. Finally, conclusions are drawn in Section 5.

Notions: The superscripts ${\left(\cdot \right)}^{T}$, ${\left(\cdot \right)}^{H}$, ${\left(\cdot \right)}^{*}$ and ${\left(\cdot \right)}^{+}$ represent the operations of transpose, Hermitian transpose, conjugate and pseudo-inverse, respectively; ⊗ stands for the Kronecker product. ⊙ stands for the Khatri-Rao product; real(·) and imag(·) are to extract the real part and imaginary part, respectively; and E(·) and angle(·) are to get the expectation and phase angle, respectively.

## 2. Problem Formulation

**Figure 1:**
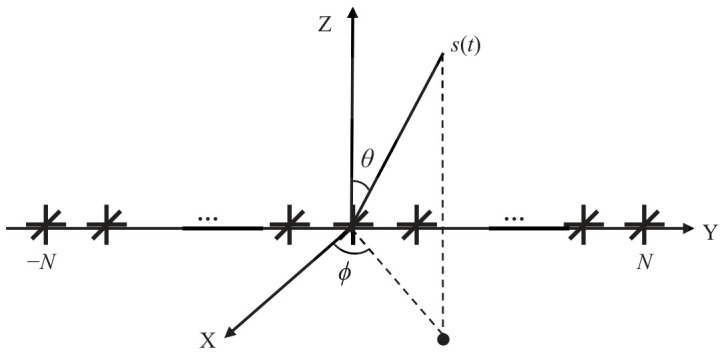
Geometry of a uniform linear tripole array.

Consider a ULA with M=2N+1 tripoles along the *y*-axis as shown in Figure 1. Each tripole consists of three co-located mutually perpendicular dipoles for measuring electric field information. Three components of each tripole are aligned with the *x*-, *y*-, and *z*-axes, respectively. All tripoles are indexed as −N,…,0,…,N. The interval between adjacent array elements is set to *d* and the carrier wavelength of the arrival signals is λ.

Assuming the array is impinged by *K* narrowband, far-field fully-polarized waves (i.e., TEM waves) parameterized by ($\theta_{k}$, $\phi_{k}$, $\gamma_{k}$, $\eta_{k}$), k=1,2,…,K(K≤N). $\theta_{k}$, $\phi_{k}$, $\gamma_{k}$ and $\eta_{k}$ represent the elevation angle, azimuth angle, polarization auxiliary angle and polarization phase difference of the *k*-th signal, respectively. Assuming that some of the *K* incident signals are coherent, the first P(P≥2) signals are coherent with each other, and the remaining K−P signals are independent to other signals. The complex envelope received at sensor *n* can be expressed as:

(1) $$\begin{array}{cc} y_{n}\left(t\right) & =\sum_{k=1}^{P}a_{p,k}s_{k}\left(t\right)e^{-j\frac{2\pi d}{\lambda }n\sin\theta_{k}\sin\phi_{k}}+n_{n}\left(t\right)\\ \; & =s_{1}\left(t\right)\sum_{k=1}^{P}a_{p,k}\beta_{k}e^{-j\frac{2\pi d}{\lambda }n\sin\theta_{k}\sin\phi_{k}}\\ \; & +\sum_{k=P+1}^{K}a_{p,k}s_{k}\left(t\right)e^{-j\frac{2\pi d}{\lambda }n\sin\theta_{k}\sin\phi_{k}}+n_{n}\left(t\right) \end{array}$$

where $s_{k}\left(t\right)$ is the complex envelope of the *k*-th signal, and $n_{n}\left(t\right)$ is the additive white noise vector at sensor *n*. $a_{p,k}$ is the polarization response vector for the *k*-th incident signal. $\beta_{k}$ denotes the amplitude and phase difference between the *k*-th coherent signal and the reference signal. In the above formula, $\beta_{1}=1$. For k=2,…,P, $\beta_{k}$ takes the form $\rho_{k}e^{j∆\varphi_{k}}$. According to the geometric symmetry, the array output at time *t* is given by:

(2) $$\begin{array}{cc} y(t) & ={\left[y_{-N}^{T}\left(t\right),\ldots ,y_{0}^{T}\left(t\right),\ldots ,y_{N}^{T}\left(t\right)\right]}^{T}\\ \; & =s_{1}\left(t\right)\sum_{k=1}^{P}\beta_{k}a_{s,k}\left(\theta ,\phi \right)⊗a_{p,k}\left(\theta ,\phi ,\gamma ,\eta \right)\\ \; & +\sum_{k=P+1}^{K}a_{s,k}\left(\theta ,\phi \right)⊗a_{p,k}\left(\theta ,\phi ,\gamma ,\eta \right)s_{k}\left(t\right)+n\left(t\right)\\ \; & =\left(A_{s}⊙A_{p}\right)s\left(t\right)+n\left(t\right)=\mathrm{As}\left(t\right)+n\left(t\right) \end{array}$$

where $s\left(t\right)={\left[s_{1}\left(t\right),\ldots ,\beta_{P}s_{1}\left(t\right),s_{P+1}\left(t\right),\ldots ,s_{K}\left(t\right)\right]}^{T}$ denotes a K×1 signal vector. The correlation between signals can be arbitrary. The steering matrix A is composed of the steering vectors and can be expressed as:

(3) $$A=\left[a_{1}\left(\theta ,\phi ,\gamma ,\eta \right),\ldots ,a_{K}\left(\theta ,\phi ,\gamma ,\eta \right)\right]$$

As the *k*-th column of matrix A, $a_{k}$ has the following form:

(4) $$a_{k}=a_{s,k}\left(\theta ,\phi \right)⊗a_{p,k}\left(\theta ,\phi ,\gamma ,\eta \right)$$

where $a_{s,k}$ denotes the spatial steering vector of the *k*-th incident signal, which is formulated as:

(5) $$a_{s,k}=\left[\begin{array}{c} e^{j\frac{2\pi d}{\lambda }N\sin\theta_{k}\sin\phi_{k}}\\ \vdots \\ 1\\ \vdots \\ e^{-j\frac{2\pi d}{\lambda }N\sin\theta_{k}\sin\phi_{k}} \end{array}\right]$$

For a tripole used in the array, following the standard model [18], the polarization response vector $a_{p,k}$ can be expressed as:

(6) $$a_{p,k}=\left[\begin{array}{cc} -\sin\phi_{k} & \cos\theta_{k}\cos\phi_{k}\\ \cos\phi_{k} & \cos\theta_{k}\sin\phi_{k}\\ 0 & -\sin\theta_{k} \end{array}\right]\left[\begin{array}{c} \cos\gamma_{k}\\ \sin\gamma_{k}e^{j\eta_{k}} \end{array}\right]$$

The additive noise n(t) is a 3M×1 zero-mean white Gaussian noise vector with variance $\sigma_{n}^{2}$ which is independent of all signals. The covariance matrix of the array output is given by:

(7) $$R=E\left\{y\left(t\right)y^{H}\left(t\right)\right\}=AR_{s}A^{H}+\sigma_{n}^{2}I$$

where I is a 3M×3M identity matrix. $R_{s}$ is the covariance matrix of the source signal vector, which is defined as:

(8) $$\begin{array}{cc} R_{s} & =E\left[s\left(t\right)s^{H}\left(t\right)\right]=\left[\begin{array}{cc} R_{s1} & O\\ O & R_{s2} \end{array}\right]\\ \; & =\left[\begin{array}{cccccc} s_{1}^{2}\left(t\right) & \ldots & \beta_{P}s_{1}^{2}\left(t\right) & 0 & \ldots & 0\\ \vdots & \ddots & \vdots & \vdots & \ddots & \vdots \\ \beta_{P}s_{1}^{2}\left(t\right) & \ldots & \beta_{P}^{2}s_{1}^{2}\left(t\right) & 0 & \ldots & 0\\ 0 & \ldots & 0 & s_{P+1}^{2}\left(t\right) & \; & \;\\ \vdots & \ddots & \vdots & \; & \ddots & \;\\ 0 & \ldots & 0 & \; & \; & s_{K}^{2}\left(t\right) \end{array}\right] \end{array}$$

where $R_{s1}$ and $R_{s2}$ denote the covariance matrix of the first *P* signals and remaining K−P signals, respectively. Notice that $\mathrm{Rank}\left\{R_{s1}\right\}=1$, so $\mathrm{Rank}\left\{R_{s}\right\}=K-P+1<K$, which means that $R_{s}$ is rank deficient. Consequently, signal subspace and noise subspace cannot be correctly separated, which leads to the failure of traditional subspace-based algorithms. Hence, necessary preprocessing is required.

## 3. Proposed Algorithm

In this section, we devote to deriving the proposed algorithm. First, we develop a preprocessing step for decorrelation. Second, we describe the proposed ESPRIT-based algorithm for estimating DOA and polarization parameters. Furthermore, the ambiguity resolution and process of pair matching is explained. Finally, the computational complexity of the proposed algorithm is analyzed.

### 3.1. Decorrelation Method Based on Toplitz Matrix Reconstruction

The basic idea of Toeplitz matrix reconstruction method is to rearrange array outputs or the covariance matrix of the received signal into a smaller-dimension Toeplitz matrix, thereby obtaining a full-rank equivalent covariance matrix to address the rank-deficiency problem. Compared with conventional methods that require subarray partitioning and covariance averaging, resulting in relatively low information utilization, the MTOEP algorithm does not require the division of the array into sub-arrays and can fully exploit the cross-correlation information across all array elements. This more comprehensive use of covariance information theoretically improves estimation accuracy. Unlike traditional scalar arrays, the ideal covariance matrix of an EMVS array composed of tripoles should be a block-Toeplitz matrix. Therefore, the conventional MTOEP cannot be directly applied in our model [33]. The main challenge lies in constructing an equivalent covariance matrix that preserves the inherent data structure of the EMVS array, enabling simultaneous estimation of DOA and polarization parameters. To overcome this issue, the proposed decorrelation method constructs a block-Toeplitz matrix as an equivalent covariance matrix and is referred to as the BMTOEP, in contrast to the MTOEP method. Firstly, similar to other matrix reconstruction algorithms, we construct a 3(N+1)×(N+1) block-Toeplitz matrix $\tilde{Y}$ for each snapshot using the array output y(t):

(9) $$\tilde{Y}=\left[\begin{array}{cccc} y_{0}\left(t\right) & y_{1}\left(t\right) & \ldots & y_{N}\left(t\right)\\ y_{-1}\left(t\right) & y_{0}\left(t\right) & \ldots & y_{N-1}\left(t\right)\\ \vdots & \vdots & \ddots & \vdots \\ y_{-N}\left(t\right) & y_{-N+1}\left(t\right) & \ldots & y_{0}\left(t\right) \end{array}\right]$$

Next, to compute the cross-correlation between each sensor output and the overall array output, the matrix $R_{\tilde{Y}i}$ is constructed. Here, $R_{\tilde{Y}i}$ denotes the correlation matrix between $\tilde{Y}$ and the received signal at *i*-th sensor, where i∈[−N,N]. Let $y_{i,1}\left(t\right)$, $y_{i,2}\left(t\right)$ and $y_{i,3}\left(t\right)$ denote the outputs of dipoles aligned with the *x*-, *y*- and *z*-axes of the *i*-th sensor at time *t*, respectively. Assuming we have received *L* snapshots, $R_{\tilde{Y}i}$ has the following expression:

(10) $$\begin{array}{cc} R_{\tilde{Y}i} & =E\left[\begin{array}{ccc} \tilde{Y}\left(t\right)y_{i,1}^{*}\left(t\right) & \tilde{Y}\left(t\right)y_{i,2}^{*}\left(t\right) & \tilde{Y}\left(t\right)y_{i,3}^{*}\left(t\right) \end{array}\right]\\ \; & =\sum_{t=1}^{L}\left[\begin{array}{ccc} \tilde{Y}\left(t\right)y_{i,1}^{*}\left(t\right) & \tilde{Y}\left(t\right)y_{i,2}^{*}\left(t\right) & \tilde{Y}\left(t\right)y_{i,3}^{*}\left(t\right) \end{array}\right] \end{array}$$

If there is only one sensor selected for the calculation of cross-correlation, it is essentially the same as the ESPRIT-like algorithm [35]. Meanwhile, if the selected array element is not sensor 0, noise components will appear off the main diagonal of matrix $R_{\tilde{Y}i}$, so that the signal subspace is mixed with noise energy. This will make it necessity to have an additional denoising process before separating the signal subspace.

It has been proved in [33] that if all sensors are selected to calculate the cross-correlation (i.e., using the cross-correlation information between all sensors to construct a covariance matrix), we obtain the equivalent covariance matrix that can be directly used for eigen-decomposition to separate the signal subspace without requiring a denoising process. So, in the proposed decorrelation method, the equivalent full-rank covariance matrix $\tilde{R}$ can be calculated by summing the squares of $R_{\tilde{Y}i}$ for i∈[−N,N].

(11) $$\tilde{R}=\sum_{i=-N}^{N}R_{\tilde{Y}i}R_{\tilde{Y}i}^{H}$$

As the equivalent covariance matrix, $\tilde{R}$ can be rewritten as:

(12) $$\tilde{R}=\tilde{A}{\tilde{R}}_{s}{\tilde{A}}^{H}+\left(N+1\right)\sigma_{n}^{4}I_{3(N+1)}$$

where $I_{3(N+1)}$ is a 3(N+1)×3(N+1) identity matrix. Matrix $\tilde{A}$ is composed of equivalent steering vector $\tilde{a}={\tilde{a}}_{s}⊗a_{p}$, with ${\tilde{a}}_{s}={\left(1,\ldots ,e^{-j2\pi Nd/\lambda \sin\theta \sin\phi }\right)}^{T}$. ${\tilde{R}}_{s}$ is a positive definite matrix with full-rank *K* whether incident signals are coherent or incoherent. The $\sigma_{n}^{4}$ term arises from higher-order noise products during the expansion of Equation (11). A detailed derivation is provided in Appendix A.

### 3.2. ESPRIT-Based Algorithm for DOA and Polarization Parameter Estimation

After performing the eigen-decomposition of $\tilde{R}$, we can obtain *K* large eigenvalues and signal subspace $E_{s}$, which is formed by their corresponding eigenvectors. According to [36], it can be shown that $E_{s}$ spans the same subspace as $\tilde{A}$, i.e., there exists a full-rank matrix $T\in C^{K\times K}$ that satisfies:

(13) $$E_{s}=\tilde{A}T$$

The directions of the signals and their polarization parameters are estimated using the signal subspace $E_{s}$. For a ULA composed of tripoles, all dipoles in a tripole have the same spatial phase difference. Therefore, the spatial steering vector of the k-th signal satisfies the shift-invariance property:

(14) $$a_{s,k}\left(2:N+1\right)=a_{s,k}\left(1:N\right){∆}_{k}$$

where

(15) $${∆}_{k}=\exp\left(-j\frac{2\pi d}{\lambda }\sin\theta_{k}\sin\phi_{k}\right)$$

Stacking all *K* signals yields the matrix relation:

(16) $$A_{2}=A_{1}∆$$

where $A_{1}$ and $A_{2}$ are the 3N×K submatrices of $\tilde{A}$, with $A_{1}$ formed by the first 3N rows and $A_{2}$ formed by rows 4 through 3(N+1). ∆ is a diagonal matrix whose *k*-th diagonal element is ${∆}_{k}$. Thus, the direction cosine of the *k*-th incident signal can be obtained from the first application of the ESPRIT algorithm.

Moreover, for each incident signal, the three orthogonal dipoles of a tripole measure the same electric field projected onto different axes. Consequently, the polarization steering vectors satisfy the proportional relations:

(17) $$\left\{\begin{array}{cc} a_{p,k}^{\left(x\right)} & =\Phi_{k}\;a_{p,k}^{\left(z\right)},\\ a_{p,k}^{\left(y\right)} & =\Lambda_{k}\;a_{p,k}^{\left(z\right)} \end{array}\right.$$

where $\Phi_{k}$ and $\Lambda_{k}$ depend solely on the DOA and polarization parameters. According to Equation (6), $\Phi_{k}$ and $\Lambda_{k}$ can be inferred as follows:

(18) $$\Phi_{k}=\frac{-\sin\phi_{k}\cos\gamma_{k}+\cos\theta_{k}\cos\phi_{k}\sin\gamma_{k}e^{j\eta_{k}}}{-\sin\theta_{k}\sin\gamma_{k}e^{j\eta_{k}}}$$

(19) $$\Lambda_{k}=\frac{\cos\phi_{k}\cos\gamma_{k}+\cos\theta_{k}\sin\phi_{k}\sin\gamma_{k}e^{j\eta_{k}}}{-\sin\theta_{k}\sin\gamma_{k}e^{j\eta_{k}}}$$

The ESPRIT algorithm is applied again by treating the dipoles aligned with the *x*-, *y*-, and *z*-axes as subarrays 1, 2, and 3, respectively. To facilitate the estimation of polarization parameters, the matrix $\tilde{A}$ is transformed using an exchange matrix J to group components corresponding to the same dipole orientation. The transformed matrix is defined as:

(20) $$\bar{A}=J\tilde{A}$$

J is exchange matrix given by $J={\left(J_{1}^{T},J_{2}^{T},J_{3}^{T}\right)}^{T}$, where $J_{i}={\left[e_{i},e_{i+3},\ldots ,e_{i+3N}\right]}^{T}$ for i=1,2,3. $e_{i}$ is a 3(N+1)×1 unit vector whose *i*-th component is 1 and the others are zero. The matrix $\bar{A}$ can be interpreted as a steering matrix with steering vector $\bar{a}=a_{p}⊗{\tilde{a}}_{s}$. Let ${\bar{A}}_{1}$, ${\bar{A}}_{2}$ and ${\bar{A}}_{3}$ be the (N+1)×K submatrices of $\bar{A}$, formed from its first, middle and last N+1 rows, respectively. Similarly, the following relationships hold:

(21) $$\left\{\begin{array}{cc} {\bar{A}}_{1} & ={\bar{A}}_{3}\;\Phi ,\\ {\bar{A}}_{2} & ={\bar{A}}_{3}\;\Lambda \end{array}\right.$$

where Φ and Λ are diagonal matrices, whose *k*-th diagonal elements correspond to $\Phi_{k}$ and $\Lambda_{k}$, respectively. Therefore, the rotation-invariant factors can be calculated using the second application of the ESPRIT algorithm.

Let $E_{s1}$ and $E_{s2}$ be the 3N×K submatrices formed from $E_{s}$, in the same manner as $A_{1}$ and $A_{2}$ are formed from $\tilde{A}$. Then, the diagonal elements of ∆ can be obtained as the eigenvalues of the matrix $\tilde{∆}=T^{-1}∆T$, which satisfies

(22) $$E_{s2}=E_{s1}\tilde{∆}.$$

In the presence of noise, $\tilde{∆}$ is estimated by solving the Least Squares (LS) problem:

(23) $$\min_{\tilde{∆}}{∥E_{s2}-E_{s1}\tilde{∆}∥}_{F}^{2}.$$

The LS solution is:

(24) $$\tilde{∆}=E_{s1}^{+}E_{s2}.$$

By left-multiplying both sides of the Equation (13) by J, we obtain the transformed signal subspace ${\bar{E}}_{s}=\bar{A}T$. Similarly, ${\bar{E}}_{s1}$, ${\bar{E}}_{s2}$, and ${\bar{E}}_{s3}$ are formed from ${\bar{E}}_{s}$ in the same manner as ${\bar{A}}_{i}$, i=1,2,3. For the polarization-domain rotation-invariant relations, the matrices are obtained by solving the LS problems

(25) $$\left\{\begin{array}{c} \min_{\tilde{\Phi }}\;{∥{\bar{E}}_{s1}-{\bar{E}}_{s3}\tilde{\Phi }∥}_{F}^{2},\\ \min_{\tilde{\Lambda }}\;{∥{\bar{E}}_{s2}-{\bar{E}}_{s3}\tilde{\Lambda }∥}_{F}^{2} \end{array}\right.$$

where $\tilde{\Phi }=T^{-1}\Phi T$, $\tilde{\Lambda }=T^{-1}\Lambda T$. The solutions are

(26) $$\tilde{\Phi }={\bar{E}}_{s3}^{+}{\bar{E}}_{s1}$$

(27) $$\tilde{\Lambda }={\bar{E}}_{s3}^{+}{\bar{E}}_{s2}$$

By performing eigen-decomposition on Equations (24), (26) and (27), all three rotation-invariant factors can be achieved.

Next, we shall derive expressions for the DOA and polarization parameters. Simplify Equations (18) and (19),

(28) $$\frac{\sin\phi_{k}}{\sin\theta_{k}\tan\gamma_{k}}e^{-j\eta_{k}}=\frac{\Phi_{k}\tan\theta_{k}+\cos\phi_{k}}{\tan\theta_{k}}$$

(29) $$\frac{\cos\phi_{k}}{\sin\theta_{k}\tan\gamma_{k}}e^{-j\eta_{k}}=\frac{\Lambda_{k}\tan\theta_{k}+\sin\phi_{k}}{\tan\theta_{k}}$$

then divide Equation (28) by Equation (29); we have

(30) $$\tan\phi_{k}=\frac{\Phi_{k}\tan\theta_{k}+\cos\phi_{k}}{\Lambda_{k}\tan\theta_{k}+\sin\phi_{k}}$$

Notice in Equation (30), the left side of the equals is a real number, but the numerator and denominator on the right side of the equation are both complex numbers. According to the rules of complex operations, when the ratio of two complex numbers is equal to a real number, the ratio of their real parts is equal to the ratio of their imaginary parts. Thus,

(31) $$\begin{array}{cc} \tan\phi_{k} & =-\frac{\mathrm{real}(\Phi_{k})\tan\theta_{k}+\cos\phi_{k}}{\mathrm{real}(\Lambda_{k})\tan\theta_{k}+\sin\phi_{k}}\\ \; & =-\frac{\mathrm{imag}(\Phi_{k})\tan\theta_{k}}{\mathrm{imag}(\Lambda_{k})\tan\theta_{k}} \end{array}$$

The azimuth angle can be calculated by

(32) $$\phi_{k}=-\arctan\left(\frac{\mathrm{imag}(\Phi_{k})}{\mathrm{imag}(\Lambda_{k})}\right)$$

The elevation angle can be calculated by

(33) $$\theta_{k}=\arcsin\left(\frac{\mathrm{angle}({∆}_{k})}{2\pi d\sin\phi_{k}}\right)$$

The *k*-th source’s polarization parameters can be estimated as

(34) $$\gamma_{k}=\arctan\left(\left|\frac{\sin\phi_{k}\tan\theta_{k}}{\left(\Phi_{k}\tan\theta_{k}+\cos\phi_{k}\right)\sin\theta_{k}}\right|\right)$$

(35) $$\eta_{k}=\mathrm{angle}\left(\frac{\sin\phi_{k}\tan\theta_{k}}{\left(\Phi_{k}\tan\theta_{k}+\cos\phi_{k}\right)\sin\theta_{k}}\right)$$

### 3.3. Ambiguity Resolution and Pair Matching

Due to the value range of the anti-trigonometric function, azimuth angle ϕ calculated by Equation (32) satisfies $\phi \in \left(-\pi /2,\pi /2\right)$, while the actual value range of ϕ is $\left(-\pi ,\pi \right)$. As a result, there exists quadrant ambiguity in the DOA estimation. For the elevation angle $\theta \in \left(0,\pi /2\right)$, sinθ is always positive. Notice in Equation (15), for azimuth angle $\phi \in \left(-\pi ,0\right)$, sinϕ is always negative. So, we can determine the quadrant of the incident angle and perform angle compensation according to the symbol of sinθsinϕ. The summary of ambiguity resolution is shown in Table 1.

So far, the elevation and azimuth angles of the incident signals have been estimated. Next, the parameter pairing procedure is introduced. The proper grouping of eigenvalues ${∆}_{k}$, $\Phi_{k}$, and $\Lambda_{k}$ is carried out in two steps. First, the eigenvalues ${∆}_{k}$ and $\Phi_{k}$ are paired according to the ordering of ${∆}_{k}$; then, ${∆}_{k}$ and $\Lambda_{k}$ are paired in the same manner. Ideally, the same eigenvector matrix *T* can be obtained through three eigen-decompositions. However, in practice, each eigen-decomposition is performed independently, which leads to column ambiguity in the resulting eigenvector matrix. To pair eigenvalues ${∆}_{k}$ and $\Phi_{k}$, assume $T_{1}$ and $T_{2}$ are the eigenvector matrices obtained from a certain eigen-decomposition of $\tilde{∆}$ and $\tilde{\Phi }$, respectively. Construct a sorting matrix $G_{1}$ as

(36) $$G_{1}=T_{2}^{H}T_{1}$$

The corresponding column vectors in $T_{1}$ and $T_{2}$ are highly correlated due to the invariance of incident signals. Therefore, the order of the corresponding columns in Φ and ∆ can be adjusted according to the matrix coordinates of the elements with the maximum absolute value in each column of $G_{1}$. Assume $T_{3}$ is a certain eigenvector matrix obtained by eigen-decomposition of $\tilde{\Lambda }$. Similarly,

(37) $$G_{2}=T_{3}^{H}T_{1}$$

**Figure 2:**
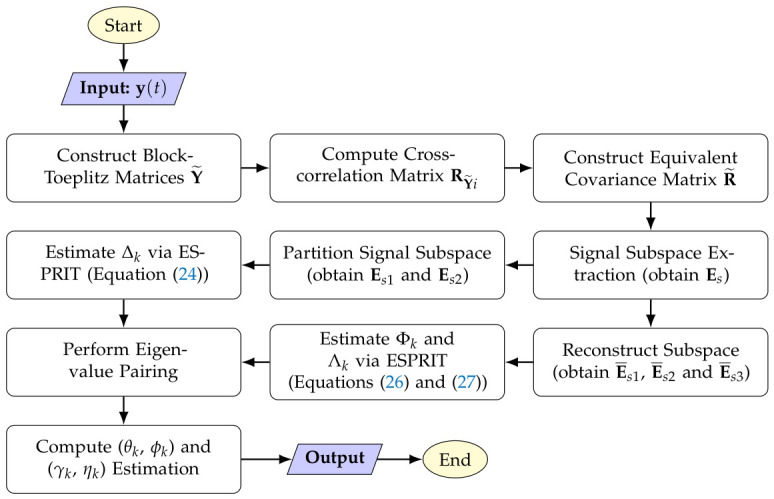
Flowchart of the proposed algorithm.

The order of the corresponding columns in $\tilde{∆}$ and $\tilde{\Lambda }$ can be adjusted based on $G_{2}$. Consequently, the columns in $\tilde{∆}$ and $\tilde{\Lambda }$ can be paired in the same manner as those in $\tilde{∆}$ and $\tilde{\Phi }$. From the paired sets of eigenvalues (${∆}_{k}$, $\Phi_{k}$, $\Lambda_{k}$), the parameter groupings ($\theta_{k}$, $\phi_{k}$, $\gamma_{k}$, $\eta_{k}$), k=1,2,…,K, can be determined. The overall procedure of the proposed algorithm is illustrated in Figure 2.

### 3.4. Complexity Analysis

In this part, we analyze the computational complexity of the proposed algorithm. The main procedure taken into account includes covariance matrix construction, eigen-decomposition, LS calculation and parameter matching. The number of vector sensors is *M* (assumed to be odd), and the number of incident signals is *K*. The construction of the block-Toeplitz matrix for both the proposed algorithm and the ESPRIT-like method requires a computational complexity of $O\left[3{\left(\frac{M+1}{2}\right)}^{2}L\right]$. *L* denotes the number of snapshots. The computational complexity of the ESPRIT-like algorithm for correlation matrix computation is $O\left[9{\left(\frac{M+1}{2}\right)}^{2}L+27{\left(\frac{M+1}{2}\right)}^{3}\right]$. In contrast, since the proposed algorithm constructs multiple block-Toeplitz matrices for all array elements, its computational complexity becomes $O\left[9M{\left(\frac{M+1}{2}\right)}^{2}L+27M{\left(\frac{M+1}{2}\right)}^{3}\right]$. Therefore, the total computational complexity for covariance matrix reconstruction in the proposed method can be approximated as $O\left[\frac{9}{4}{\left(M+1\right)}^{3}L+\frac{27}{8}{\left(M+1\right)}^{3}M\right]$. For comparison, the computational complexity of constructing the covariance matrix using forward spatial smoothing (FSS) method is $O\left[9M^{2}L+9{\left(\frac{M+1}{2}\right)}^{3}\right]$. In the following ESPRIT-based algorithm, the computational complexity of the eigen-decomposition is $O\left[{\left(\frac{3(M+1)}{2}\right)}^{3}+3K^{3}\right]$. LS calculation requires $O\left[6\left(\frac{M-1}{2}\right)K^{2}+4\left(\frac{M+1}{2}\right)K^{2}\right]$. The parameter matching process requires around $O\left[2(K^{3}+K^{2})\right]$. The comparison of total computational complexity is shown in Table 2.

## 4. Simulation Results

In this section, we conduct numerical simulations to verify the effectiveness of our proposed method in terms of root-mean-square error (RMSE). The average RMSE is defined as:

(38) $$RMSE_{\mathrm{DOA}}=\frac{1}{K}\sum_{k=1}^{K}\sqrt{\frac{1}{W}\sum_{w=1}^{W}{\left({\hat{\theta }}_{k,w}-\theta_{k}\right)}^{2}+{\left({\hat{\phi }}_{k,w}-\phi_{k}\right)}^{2}}$$

where *W* is the replication number of Monte Carlo runs, and ${\hat{\theta }}_{k,w}$ is the estimation of $\theta_{k}$ in the *w*-th Monte Carlo trial. It is considered a successful resolution when the spatial angle error is less than 1°. For simplicity, we suppose that the power of all signal sources equals $\sigma_{s}^{2}$, and the signal-to-noise ratio (SNR) is defined as $10\log_{10}\left(\sigma_{s}^{2}/\sigma_{n}^{2}\right)$. In all simulations, consider two coherent TEM signals impinging on a ULA composed of seven tripoles with d=λ/2. Each incident signal is assumed to be fully elliptically polarized. The polarization state is characterized by the auxiliary polarization angle γ and the phase difference η, as defined in Section 2. In the simulations, the polarization parameters of each source are fixed when generating the received data.

### 4.1. Effectiveness of the Algorithm

**Figure 3:**
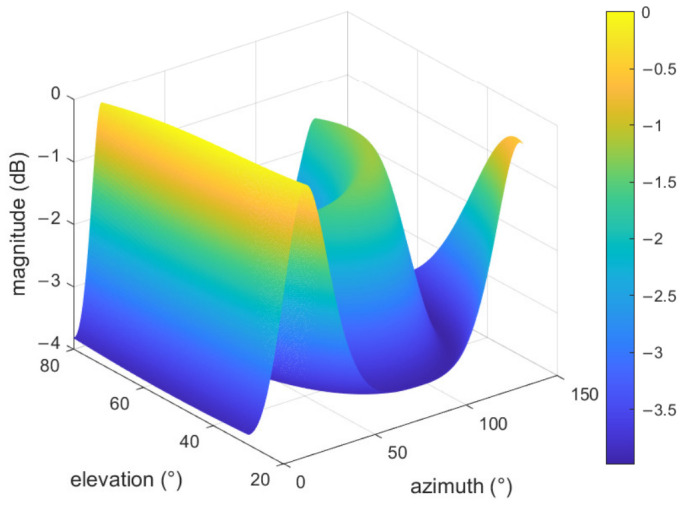
DOA spectrum with the polarized-MUSIC algorithm.

To validate the effectiveness of the proposed algorithm, both polarized-MUSIC and the proposed algorithm are applied under identical conditions. Two incident signals are parameterized by $\left(\theta_{1},\phi_{1},\gamma_{1},\eta_{1}\right)=\left(45{}^{\circ},20{}^{\circ},30{}^{\circ},60{}^{\circ}\right)$, $\left(\theta_{2},\phi_{2},\gamma_{2},\eta_{2}\right)=\left(60{}^{\circ},120{}^{\circ},45{}^{\circ},-60{}^{\circ}\right)$, respectively. The number of snapshots is 500, and the SNR is set to 15 dB. A total of 100 Monte Carlo experiments are performed. According to the simulation results shown in Figure 3, multiple spurious peaks and a broadened main lobe appear in the polarized-MUSIC spatial spectrum due to the coherency between incident signals, making it impossible to distinguish coherent sources. This indicates that decorrelation processing is necessary before applying subspace-based methods for parameter estimation.

**Figure 4:**
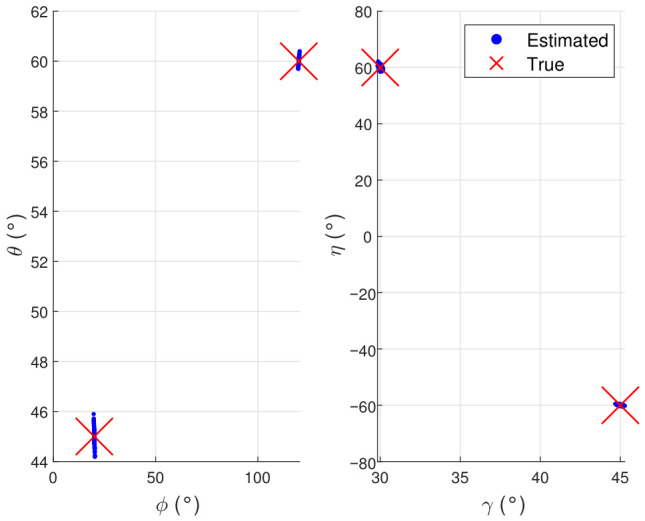
Scatter results of the proposed method.

In Figure 4, the estimated DOA–polarization pairs form two compact clusters around the true values, and no noticeable outliers are observed, showing that both directions and polarization parameters have been identified and paired successfully with high estimation accuracy.

### 4.2. RMSE Performance Versus SNR and Number of Snapshots

**Figure 5:**
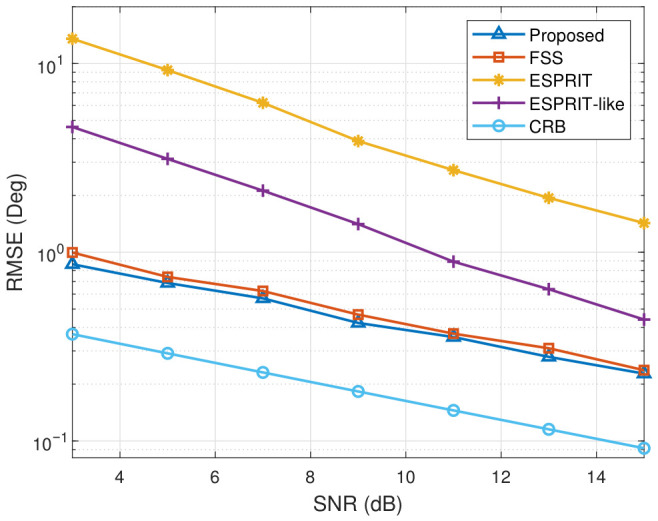
Average RMSE of DOA estimation versus SNR.

In this part, we design experiments to test the accuracy of DOA estimation. To evaluate the performance of multiple parameter estimations, the average RMSE is used as the performance metric. First, we investigate the RMSE performance of three decorrelation preprocessing methods (ESPRIT-like [35], FSS [19], and the proposed BMTOEP algorithm), as well as the case where the ESPRIT algorithm is directly applied without any decorrelation preprocessing, with respect to SNR. The ESPRIT-like method represents a low-complexity decorrelation approach using a single reference sensor. The Cramér–Rao Lower Bound (CRB) [37] is also included as a benchmark in the simulation results. It should be noted that the CRB is obtained via numerical evaluation based on the adopted data model, rather than a closed-form analytical derivation. The same signal parameters as in Section 4.1 are adopted, while the SNR varies from 3 to 15. The predetermined number of snapshots is 1000. After 100 Monte Carlo trials, the RMSE of DOA estimation for two coherent signals is illustrated in Figure 5.

As shown in Figure 5, the RMSE of all methods decreases as SNR increases. The method without decorrelation preprocessing is ineffective due to its significant errors. The performance of the ESRPIT-like algorithm is poor and becomes effective only when the signal-to-noise ratio exceeds 10 dB. Besides, the performance gap between ESPRIT-like and the CRB gradually decreases as SNR increases. It can also be observed that the proposed method maintains a smaller gap to the CRB and produces slightly lower RMSE than the FSS method across the entire SNR range. This is because FSS uses only the autocorrelation information on the principal diagonal of the signal covariance matrix to obtain an equivalent covariance matrix. In contrast, the proposed method utilizes the total information to reconstruct the equivalent source covariance matrix.

**Figure 6:**
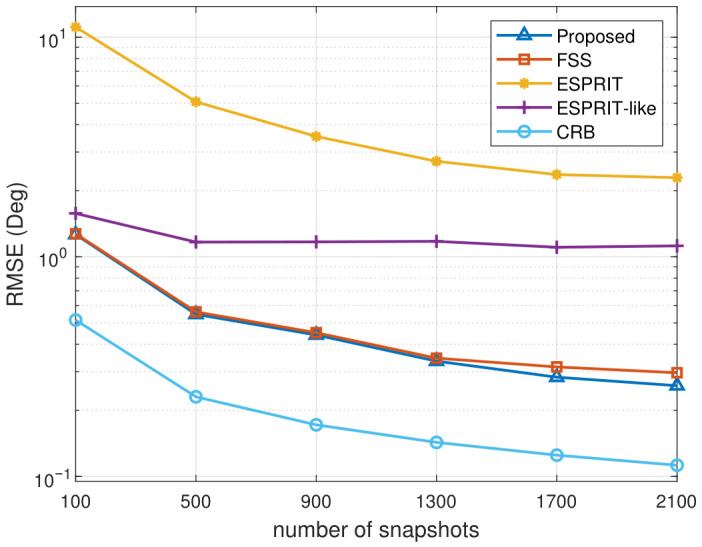
Average RMSE of DOA estimation versus number of snapshots.

To further assess the performance of the proposed algorithm, we compare the DOA estimation RMSE versus the number of snapshots. Figure 6 illustrates the comparison of RMSE curves for different algorithms as the number of snapshots varies. The simulation conditions are the same as above, except that SNR is fixed at 15 dB and the number of snapshots varies between 100 and 2100.

In Figure 6, the RMSE of all methods generally decreases as the number of snapshots increases, indicating improved estimation accuracy with more available data. It can be observed that the ESPRIT-like method exhibits a performance saturation when the number of snapshots exceeds 500. This is mainly attributed to the inherent limitations of the decorrelation strategy adopted in that method, where the available array information is not fully utilized, and thus increasing the number of snapshots does not further improve the estimation accuracy. It also demonstrates that the proposed method consistently achieves the lowest RMSE across the entire snapshot range, while FSS exhibits slightly higher errors but follows a similar downward trend. The method without decorrelation remains inaccurate regardless of the number of snapshots.

### 4.3. RMSE Performance Versus Angular Separation

To assess the resolution and probability of successful estimation, we evaluate the algorithm performance and success probability based on angular separation between two coherent signals. The azimuth angles of both signals are identical and fixed at 100°. The pitch angle of signal 1 is fixed at 30°. The polarization parameters (γ,η) of signal 1 and signal 2 are (20°, −80°) and (50°, 80°), respectively. The spatial separation angle between the two signals varied from 2° to 14° by changing the pitch angle of signal 2. A total of 100 Monte Carlo trials are performed with an SNR fixed at 15 dB, and the number of snapshots is set to 1000.

**Figure 7:**
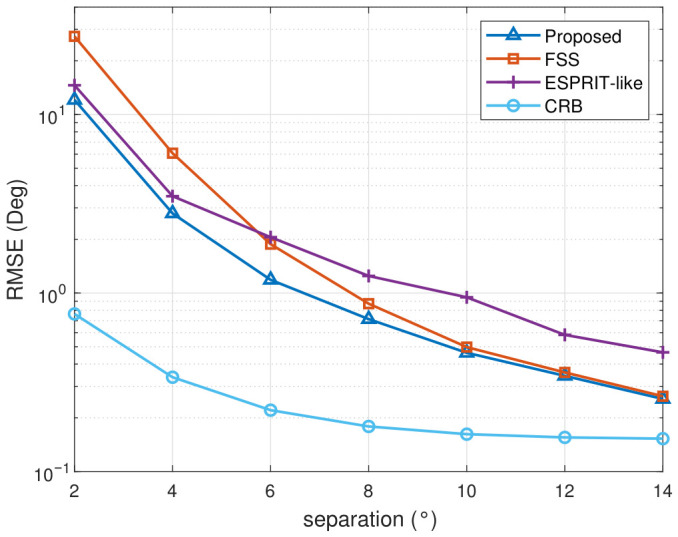
Average RMSE versus angular separation.

**Figure 8:**
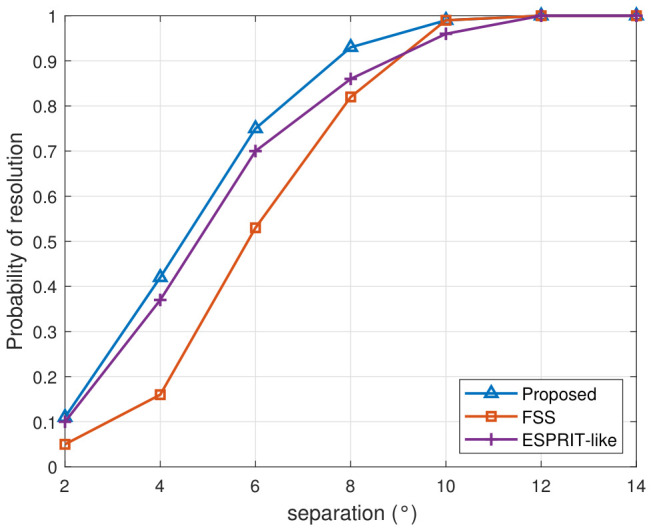
Probability of resolution versus angular separation.

As shown in Figure 7 and Figure 8, both the DOA estimation accuracy and the probability of success improve as the angular separation between the two coherent sources increases. Compared with the CRB, the proposed method exhibits the smallest performance gap across the entire separation range. When the separation is below 4°, the RMSE curves of the proposed method and ESPRIT-like method are close to each other. As the separation grows, the proposed method maintains the lowest RMSE, while the gap between the proposed method and FSS gradually narrows. Moreover, the proposed method approaches CRB more closely as the separation grows because the steering vectors of the two sources become more distinguishable. For sufficiently large separations, the proposed method and FSS tend to achieve comparable estimation accuracy, while the ESPRIT-like retains noticeably higher errors.

Figure 8 shows that the probability of successful resolution increases with angular separation. The proposed method achieves the highest success rate when the separation is small. All algorithms achieve a 100% success rate when the spatial separation angle exceeds 12°. These results suggest that the proposed method has advantages in scenarios where coherent sources are closely spaced.

### 4.4. Computational Complexity Versus Number of Sensors

**Figure 9:**
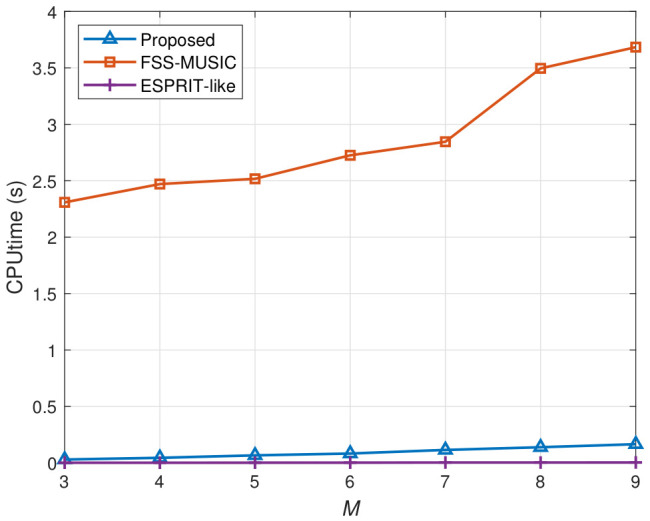
CPU runtime versus the number of sensors.

In the last experiment, the computational burden of different algorithms is compared in terms of CPU runtime. We evaluate the runtime performance of the proposed algorithm, FSS-MUSIC and the ESPRIT-like algorithm with the number of sensors *M* varying from 7 to 19. The average runtimes were obtained from a PC with an Intel(R) Core(TM) i3-7100 3.9 GHz CPU and 8 GB RAM by running Matlab (Ver. 2021a) codes for 50 independent trials. We set the number of snapshots to L=500 and the number of incident signals to K=2. For the FSS-MUSIC algorithm, the smoothing time is N+1. The comparison results in Figure 9 show that the runtime of FSS-MUSIC increases rapidly with the number of sensors and is significantly higher than that of the other two methods. This is mainly because it relies on spectral peak searching, which leads to higher computational complexity. In contrast, both the proposed algorithm and the ESPRIT-like algorithm follow the ESPRIT framework and therefore avoid the spectral search procedure, resulting in substantially lower runtime. Although the ESPRIT-like method achieves the lowest runtime, the proposed algorithm maintains a comparable computational cost while providing improved estimation performance in previous experiments. These results demonstrate that the proposed algorithm achieves a favorable trade-off between estimation accuracy and computational efficiency, making it suitable for practical engineering applications.

## 5. Discussions

From the simulation results, it can be observed that the proposed BMTOEP decorrelation method provides improved estimation accuracy and resolution compared with classical approaches. It should be noted that the proposed algorithm is developed under the ULA model and relies on the shift-invariance property of the array, which leads to a block-Toeplitz-structured covariance matrix. Nevertheless, the core idea of covariance reconstruction is not inherently restricted to ULA. The proposed method can be extended to other array configurations that preserve similar translational invariance, such as uniform rectangular arrays (URAs), where a multidimensional Toeplitz structure can be constructed. For array geometries that do not satisfy such properties (e.g., nested or coprime arrays), the proposed method may still be applicable by transforming the array into an equivalent virtual uniform array through appropriate preprocessing techniques. However, such extensions require additional processing steps and are beyond the scope of this work.

In addition, this work assumes ideal electromagnetic vector sensors and does not explicitly consider mutual coupling effects. In practical array systems, mutual coupling may distort the array manifold and degrade estimation performance. This effect can be incorporated into the signal model by introducing a mutual coupling matrix that modifies the steering vector. If the coupling matrix is known or can be accurately calibrated, its impact can be compensated accordingly. Otherwise, more advanced calibration or robust estimation techniques may be required. The investigation of mutual coupling effects and their mitigation is an important topic and will be considered in future work.

## 6. Conclusions

In this paper, an ESPRIT-based direction-finding algorithm for uniform linear tripole arrays combined with the BMTOEP decorrelation technique is proposed. By fully exploiting the complete cross-correlation information among all array outputs, the proposed method reconstructs a full-rank block-Toeplitz covariance matrix and alleviates the rank-deficiency problem without requiring a denoising process. Closed-form expressions for DOA and polarization estimation are derived within the ESPRIT framework, and a parameter-pairing strategy is introduced to guarantee the correct pairing of multidimensional parameters. The proposed algorithm avoids spectral peak searching and thus has great advantages in computational burden compared with classical MUSIC-based methods. Simulation results show that the proposed method consistently achieves lower RMSE and improved resolution than the benchmark algorithms across the considered scenarios, especially in low-SNR and small-angular-separation scenarios. In particular, compared with FSS, the quantitative results show approximately a 10% RMSE reduction at a low SNR of 3 dB and an improvement of about 12 percentage points in success rate at an angular separation of 8°. Additionally, the estimation error is reduced by about 16%. Future work will focus on integrating mutual coupling modeling into the proposed framework, and validating the algorithm with real-world measurement data to enhance its applicability in practical array systems.

## Figures and Tables

**Table 1:** All cases of ambiguity resolution.

	tanϕ>0	tanϕ<0
sinθsinϕ>0	$\theta_{new}=\theta$	$\theta_{new}=-\theta$
	$\phi_{new}=\phi$	$\phi_{new}=\pi +\phi$
sinθsinϕ<0	$\theta_{new}=-\theta$	$\theta_{new}=\theta$
	$\phi_{new}=\phi -\pi$	$\phi_{new}=\phi$

**Table 2:** Comparison of computational complexity among the three algorithms.

Algorithm	Total Computational Complexity
BMTOEP	$O\left[\frac{9}{4}{\left(M+1\right)}^{3}L+\frac{27}{8}{\left(M+1\right)}^{4}+\left(5M+1\right)K^{2}+5K^{3}\right]$
ESPRIT-like	$O\left[3{\left(M+1\right)}^{2}L+\frac{27}{4}{\left(M+1\right)}^{3}+\left(5M+1\right)K^{2}+5K^{3}\right]$
FSS	$O\left[9M^{2}L+\frac{9}{2}{\left(M+1\right)}^{3}+\left(5M+1\right)K^{2}+5K^{3}\right]$

## Data Availability

The original contributions presented in this study are included in the article. Further inquiries can be directed to the corresponding author.

## Appendix A. Derivation of the Noise Term in the Equivalent Covariance Matrix

In this appendix, we provide a detailed derivation of the noise term in the equivalent covariance matrix $\tilde{R}$ for the proposed BMTOEP algorithm. According to Equation (2), the array output can be expressed as:

(A1) $$y\left(t\right)=y_{s}\left(t\right)+n\left(t\right)$$

The noise vector at the *k*-th sensor is denoted as $n_{k}\in C^{3\times 1}$, which is assumed to be complex additive white Gaussian noise (AWGN) with:

(A2) $$E\left[n_{i}n_{j}^{H}\right]=\sigma_{n}^{2}I_{3}\delta_{ij}$$

where $\delta_{ij}$ is the Kronecker delta. The reconstructed matrix $\tilde{Y}$ has:

(A3) $$\tilde{Y}={\tilde{Y}}_{s}+\tilde{N}$$

To represent the block-Toeplitz structure of the reconstructed noise matrix $\tilde{N}\in C^{3(N+1)\times (N+1)}$, we define the selection matrix $D_{i}\in R^{\left(N+1)\times (N+1\right)}$ as:

(A4) $${\left[D_{i}\right]}_{k,m}=\left\{\begin{array}{cc} 1, & k-m=i\\ 0, & \mathrm{otherwise} \end{array}\right.$$

where i∈[−N,N]. Thus, the constructed noise matrix $\tilde{N}$ can be expressed as:

(A5) $$\tilde{N}=\sum_{j=-N}^{N}D_{j}⊗n_{j}$$

The correlation matrix between the reconstructed noise matrix $\tilde{N}$ and the noise vector of the *i*-th sensor $n_{i}$ is given by:

(A6) $$R_{ni}=E\left[\tilde{N}n_{i}^{H}\right]=E\left[\left(\sum_{j=-N}^{N}D_{j}⊗n_{j}\right)n_{i}^{H}\right]$$

Utilizing the property of the Kronecker product (A⊗B)C=A⊗(BC), we have:

(A7) $$R_{ni}=\sum_{j=-N}^{N}D_{j}⊗E\left[n_{j}n_{i}^{H}\right]=D_{i}⊗\left(\sigma_{n}^{2}I_{3}\right)=\sigma_{n}^{2}\left(D_{i}⊗I_{3}\right)$$

The noise component of the equivalent covariance matrix is defined as the summation of the squared correlation matrices:

(A8) $${\tilde{R}}_{noise}=\sum_{i=-N}^{N}R_{ni}R_{ni}^{H}=\sum_{i=-N}^{N}\left[\sigma_{n}^{2}\left(D_{i}⊗I_{3}\right)\right]{\left[\sigma_{n}^{2}\left(D_{i}⊗I_{3}\right)\right]}^{H}$$

Applying the property (A⊗B)(C⊗D)=(AC)⊗(BD), the expression simplifies to:

(A9) $${\tilde{R}}_{noise}=\sigma_{n}^{4}\sum_{i=-N}^{N}\left(D_{i}D_{i}^{T}\right)⊗\left(I_{3}I_{3}^{H}\right)=\sigma_{n}^{4}\left(\sum_{i=-N}^{N}D_{i}D_{i}^{T}\right)⊗I_{3}$$

Note that $D_{i}D_{i}^{T}$ is a diagonal matrix where the *k*-th diagonal element is 1 if the *i*-th diagonal of $D_{i}$ exists at row *k*. Since the summation $\sum_{i=-N}^{N}D_{i}D_{i}^{T}$ covers all possible diagonals for each row k∈[0,N], and each row in the original N+1 sensors contributes exactly once to each block row, we obtain:

(A10) $$\sum_{i=-N}^{N}D_{i}D_{i}^{T}=\left(N+1\right)I_{N+1}$$

Substituting this back into the expression for ${\tilde{R}}_{noise}$, we arrive at the final result:

(A11) $${\tilde{R}}_{noise}=\sigma_{n}^{4}\left[\left(N+1\right)I_{N+1}⊗I_{3}\right]=\left(N+1\right)\sigma_{n}^{4}I_{3(N+1)}$$
